# Development of a Quinoa-Based Fermentation Medium for Propagation of *Lactobacillus Plantarum* and *Weissella Confusa* in Opaque Beer Production

**DOI:** 10.1155/ijm/5745539

**Published:** 2025-01-21

**Authors:** Shepherd Manhokwe, Tatenda Musarurwa, Talknice Z. Jombo, Desmond T. Mugadza, Amiel Mugari, Joseph Bare, Scelo Mguni, Fidelis Chigondo, Jane Tafadzwa Muchekeza

**Affiliations:** ^1^Department of Food Science and Nutrition, Midlands State University, P Bag 9055, Gweru, Zimbabwe; ^2^Department of Applied Biosciences and Biotechnology, Midlands State University, P Bag 9055, Gweru, Zimbabwe; ^3^Department of Chemical Sciences, Midlands State University, P Bag 9055, Gweru, Zimbabwe; ^4^Department of Animal and Wildlife Sciences, Midlands State University, P Bag 9055, Gweru, Zimbabwe

**Keywords:** lactic acid bacteria, medium development, quinoa, starter culture

## Abstract

Product inconsistency of opaque beer has for long been a tenacious problem in the brewing industry since the current process relies on spontaneous lactic acid fermentation. In order to impede this challenge, there is a need to add lactic acid bacteria (LAB) starter cultures in opaque beer brewing to improve its organoleptic qualities. This study sought to develop a quinoa-based fermentation medium for propagation of *Lactobacillus plantarum* and *Weissella confusa* as potential starter cultures in opaque beer production. An evaluation of the stability and tolerance of the LAB under various stress conditions was also done. Fermentation wort from opaque beer brewing and different quinoa-based synthetic media with varying nutritional components was prepared for propagation of LAB. Physiochemical analyses which included pH, Brix value and total titratable acidity (TTA) of monocultured and cocultured synthetic media were measured. The measurements were done at 24 h time intervals ranging from 0 to 96 h. Tolerance studies which included the effect of heat shock, cold shock, oxidative stress and osmotic pressure on the survival rate of LAB were conducted to determine the stability of LAB. MRS with *L. plantarum* monoculture (MRSp) had a notable change in pH from 4.5 to 3.6 after 24 h. The cocultured (M5p + w) synthetic media and cocultured MRS (MRSp + w) also exhibited change in pH from 4.3 to 3.2 and 4.3 to 3.3, respectively, after 72 h. Brix value in all media samples decreased after 24 h except for the uninoculated MRS sample (MRS C). The synthetic and coculture medium (M5p + w) exhibited an increase in TTA (0.79% (m/v) lactic acid) within the first 24 h. Exposure to heat shock had a significance effect (*p* < 0.05) on the survival rate of *L. plantarum* and *W. confusa*. The *W. confusa* in synthetic media recorded a higher survival rate (27 ± 0.03%) upon exposure to heat shock than *L. plantarum* (7 ± 0.01%). In contrast, *L. plantarum* in MRS recorded a higher survival rate (67 ± 0.02%) upon exposure to cold shock and oxidative stress (34 ± 0.01%). The starter cultures tested survived upon exposure to the stress conditions, indicating their potential use in opaque beer production.

## 1. Introduction

Opaque beer is a traditional and popular beverage in several countries in Africa [1]. The beer is made from maize grits, sorghum malt, barley malt, water, lactic acid (LA) and a top-fermenting strain of the yeast (*Saccharomyces cerevisiae*) [2]. The brewing process, involving both LA fermentation and alcoholic fermentation, takes about 5–7 days. The major biological changes occurring in the brewing process are catalysed by natural lactic acid bacteria (LAB) and *S*. *cerevisiae*, introduced as a starter culture [3]. Both yeast and LAB can add to the organoleptic profile by producing a variety of flavour compounds during fermentation. In the production of opaque beer, the main metabolite released by LAB is LA, which creates the specific sour taste that contributes to the “bite” of the beer. The sour beer is characterised by a low ethanol content (approximately 4.5%) and the fermentation with LAB is considered to be an effective way to improve flavour. Flavour is one of the important factors that affect the sensory quality and determines the acceptability and preference of products [4, 5]. The current production process of opaque beer relies on spontaneous LA fermentation. This spontaneous LA fermentation is one of the contributing factors to the inconsistent quality of the beer [6]. Due to multiple microorganisms' activities, spontaneous fermentation remains difficult to control [7] and product shelf-life is compromised.

Quinoa (*Chenopodium quinoa*) originates from the Andes. Its seed contains 14%–18% protein with well-balanced amino acid composition, especially lysine, a good source of Group B vitamins, essential fatty acids, antioxidants and trace elements [8–10]. The amino acid composition is rich in essential amino acids such as lysine (5.1%–6.4%) and methionine (0.4%–3.1%) [11]. Quinoa is also known as a high source of vitamins (folate and tocopherols), minerals (iron, calcium, copper, manganese, and potassium) and other phytochemicals (ecdysteroids, phenolic acids, and flavonoids such as kaempferol and quercetin) [12–14]. Quinoa fermentation by LAB is an interesting alternative to produce new products with high nutritional value [13, 15]. Its potential application in medium development has been largely underexplored as protein quality and quantity in quinoa, are critical components in defining the nutritional properties of a fermentation media.

Microorganisms used in commercial starter cultures include bacteria, yeasts and moulds [7]. LAB are a group of bacteria that can use carbohydrates to produce LA as a main metabolite [16–18]. LAB have a safe history of use by humans, and they are responsible for the production of many valuable molecules such as organic acids, exopolysaccharides, polyols and antimicrobial compounds [17]. Lactic-fermented foods have been associated with immense health benefits, safety, nutritional and sensory improvements [7, 18–21]. Although LAB include more than 60 genera, the frequently genera occur in food fermentation generally include *Lactobacillus*, *Lactococcus*, *Leuconostoc*, *Pediococcus*, *Streptococcus*, *Enterococcus* and *Weissella* [22]. Togo, Feresu and Mutukumira [6] confirmed the presence of LAB, *Lactobacillus*, Lactococcus, Leuconostoc, Streptococcus and Enterococcus, in opaque beer. *L*. *plantarum* plays a significant role in a wide range of spontaneous and controlled lactic fermentations in food processing [23]. *L. plantarum*, for instance, is not only appropriate from a biochemical perspective but also safe to be used in food products [21, 24, 25]. Cocultures can be used in the production of fermented foods. The presence and synergistic effect of both *L. plantarum* and *Weissella confusa* in fermented products has been documented [8, 26]. *Weissella* is a genus of LAB consisting of species formerly included in the *Leuconostoc paramesenteroides* group and commonly found in fermented foods, milk, vegetables and faeces environment [27–30]. *W. confusa* has a diverse environmental distribution with broad applications. The tolerance of these LAB to stressors is critical for screening potential candidates for application as starter cultures [31].

The aim of this study was to obtain a low-cost medium for starter cultures with potential application in industrial scale opaque beer production. *L. plantarum* and *W*. *confuse* growth and stress tolerance were evaluated with a quinoa-based medium as a carbohydrate and/or nitrogen source.

## 2. Methodology

### 2.1. Isolation and Identification of LAB

LAB strains of *L. plantarum* and *W. confusa* were isolated from opaque beer samples obtained from a local Sorghum beer processing plant. Serial dilutions of the opaque beer samples up to 10^−5^ were prepared using peptone water and subcultured aseptically onto de-Man Rogosa and Sharpe (MRS) agar plates using the spread plate technique. The plates were incubated at 37°C for 24 h anaerobically to provide optimum growth conditions for LAB. Morphologically different colonies were picked from the plates and subcultured on a separate plate of MRS agar. The plates were then incubated 37°C for 72 h under anaerobic conditions. The *L. plantarum* and *W. confusa* starter cultures were positively identified on MALDI-TOF MS (Matrix-Assisted Laser Desorption Ionization-Time of Flight Mass Spectrometry) at University of Pretoria, South Africa, following the procedure described by Mugadza et al. [32]. Isolates were streaked on MRS agar and incubated for 24 h at 30°C. Samples for MALDITOF–MS analysis were prepared using ethanol treatment followed by extraction with formic acid and acetonitrile as described by Drevinek et al. [33]. They were identified using MALDI-TOF–MS software (MALDI biotyper 3.0 Bruker Daltonics) after mass spectra were obtained using a MALDI-TOF–MS (Bruker Daltonics) following a procedure by Dybwad et al. [34].

### 2.2. Preparation of Sterilised Wort (WS)

Wort filtrate was prepared by filtering (Whatman No. 1) the opaque beer collected from a local Sorghum beer processing plant. Two samples of wort filtrates were prepared. One sample was sterilized at 121°C for 15 min. The other sample was tyndallized by sterilizing at 121°C for 15 min using an autoclave (A-912X, Hi-Care USA), then cooled at 4°C to allow sporulation to take place, and then sterilizing again at 121°C for 15 min to inactivate the spores.

### 2.3. Preparation of the Synthetic Media

Quinoa was hydrolysed to provide the nutritive compounds of the medium. White quinoa grains were milled to a fine powder using an analytical mill (IKA A10 basic, Germany). Quinoa powder (8 g) was mixed with 3% hydrochloric acid in 100 mL distilled water and hydrolysed at 121°C for 30 min using an autoclave (A-912X, Hi-Care USA). Afterwards, the flask was cooled. Sodium bicarbonate (Na_2_CO_3_) was added to neutralize the acid until no fizzing was observed. The liquid was filtered using a filter paper (Whatman No. 1) to remove the granular residues. Quinoa hydrolysate, calcium carbonate, magnesium chloride, sucrose, water, whey milk and vegetable oil were mixed, as shown in Table 1, and sterilized at 121°C for 15 min using an autoclave. The cooled mixtures were filtered to produce a clear medium (synthetic media).

### 2.4. LA Fermentation

A loop of *L. plantarum (p)* or *W. confusa (w)* was aseptically transferred to 50 mL of MRS broth, WS, tyndallized wort (WT) and the synthetic media (M) as shown in Table 2. The culture was incubated for 96 h at 30°C. Cells were periodically harvested and separated by centrifuging for 5 min. The cell pellet was washed with saline solution (0.85% NaCl) and resuspended. A UV-Vis spectrophotometer (Agilent Carry 60) at 660 nm was used to measure the absorbance for cellular growth.

### 2.5. Determination of Titratable Acidity

Filtrates (10 mL) from the samples were pipetted into a glass beaker. Three drops of phenolphthalein red indicator were added to the filtrate and titrated with 0.1 M NaOH standard solution to a slight pink colour. The results were expressed in % (m/v) LA.

(1) $$\begin{array}{c} {\text{CH}}_{3}{\text{CHOHCO}}_{2}H+\text{NaOH}⟶{\text{CH}}_{3}{\text{CHOHCO}}_{2}\text{Na}+H_{2}O. \end{array}$$

LA has a relative molecular mass of 90 gmol^−1^

Hence, 1 mL of 0.1 M NaOH ≡ 0.009 g CH_3_CHOHCO_2_H.

Therefore, *x* ml of 0.1 M NaOH ≡ (*x* ml × 0.009) g CH_3_CHOHCO_2_H; therefore,

(2) $$\begin{array}{c} \text{Total titratable acidity}\left(\text{TTA}\right)\%\left(m/v\right)=x\text{ ml}\left(0.1M\text{ NaOH}\right)\times 0.09, \end{array}$$

where *x* ml is the volume of 0.1 M NaOH.

The TTA was calculated as shown in equation (2) above.

### 2.6. Determination of Brix Value

A refractometer (S-DT-016, Japan) was used to measure the sugar content of the growth media samples. It measures the specific gravity of the solution using a Brix scale. A drop of hydrolysate is placed on the refractometer and Brix is measured in (Bx %).

### 2.7. Determination of pH

The pH of the samples was measured using a pH metre (HANNA H198128). The pH metre was calibrated before use with buffers of pH 4, 7 and 10. The digital metre electrode was dipped into the 10 mL of the filtrates and pH was recorded.

## 3. Tolerance Studies

### 3.1. Effect of Osmotic Pressure on the Survival Rate

After 24 h incubation at 37°C, 1 mL of inoculated medium was inoculated in MRS broth and the synthetic medium that had been treated with 25% (v/v) sodium chloride. The samples were then incubated in 37°C for 24 h. After 24 h, optical density of all samples was determined using a UV-Vis spectrophotometer at 660 nm using MRS broth as a blank.

### 3.2. Effect of Oxidative Stress on the Survival Rate

The starter cultures were inoculated in MRS broth and the synthetic media which had been treated with 0.05% L-Cysteine and L-ascorbate and then deaerated by boiling. The media samples were then incubated in 37°C for 24 h. Optical density of all the media samples was determined using a UV-Vis spectrophotometer at 660 nm using MRS broth as a blank.

### 3.3. Effect of Cold Shock on the Survival Rate

The starter cultures were inoculated in MRS broth and the synthetic media and then incubated in 37°C for 24 h. The media samples were taken after the 24 h and placed at 0°C for 24 h. The samples were exposed to room temperature, thawed and incubated again at 37°C for 24 h. Optical density of the samples was determined using a UV-Vis spectrophotometer at 660 nm using MRS broth as a blank.

### 3.4. Effect of Heat Shock on the Survival Rate

The starter cultures were inoculated in MRS broth and the synthetic media. The media samples were then incubated at 37°C for 24 h. Inoculated media were taken after the 24 h and heated at 52°C for 15 min. Afterwards, the tubes were cooled and incubated at 37°C for 24 h. The optical density of samples was determined using a UV-Vis spectrophotometer at 660 nm using MRS broth as a blank.

## 4. Results

**Figure 1:**
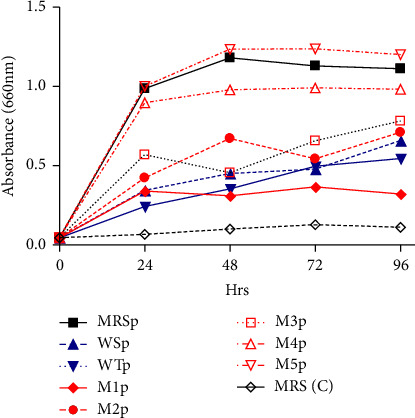
Effect of media formulation on the growth rate of *L. plantarum*.

As illustrated in Figure 1, the most significant increase in absorbance over time after inoculation is observed in synthetic medium inoculated with *L. plantarum* sample (Mp). As shown in Figure 1, absorbance increased with time, indicating an increase in the concentration of LAB over time. The MRS (C) sample, which was the MRS broth uninoculated with *L. plantarum*, showed the lowest growth rate since the absorbance remained almost levelled off as the fermentation period progressed.

**Figure 2:**
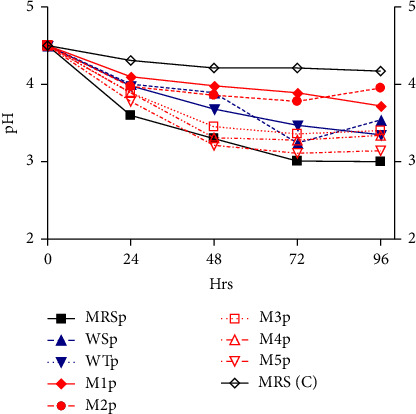
Effect of *L. plantarum* on pH in different media formulations.

As shown in Figure 2, there is decrease in pH over time of incubation. MRS inoculated with the *L. plantarum* (MRSp) had a notable change in pH from 4.5 to 3.6 after 24 h while the uninoculated MRS (MRS C) sample had the least change in pH from 4.5 to 4.2 after 96 h. The WT sample inoculated with *L. plantarum* (WTp) showed fluctuations in pH changes over time.

**Figure 3:**
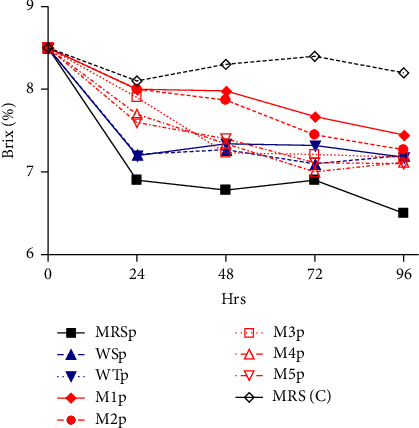
Effect of *L. plantarum* on the Brix value in different media formulations.

As shown in Figure 3, there was a notable decrease in the Brix value in all media samples after 24 h except for the uninoculated MRS sample (MRS C) which acted as the control. MRS inoculated with the culture (MRSp) had the most significant drop from a Brix value of 8.5% to 6.9% after a period of 24 h. However, the Brix values fluctuated thereafter for all samples. At 48 h, the inoculated synthetic media 1 (M1p) and 2 (M2p) samples had a slight changes in Brix values from 8.5% to 7.98% and 7.87%, respectively.

**Figure 4:**
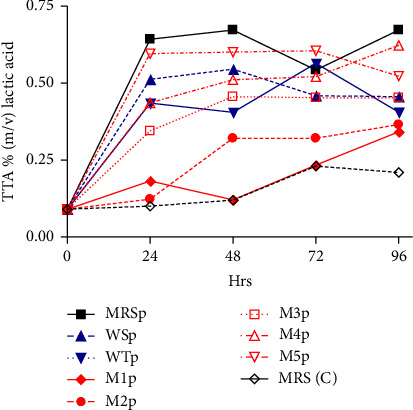
Effect of *L. plantarum* on TTA in different media formulations.

There was an increase in TTA over time after inoculation for all samples assessed (Figure 4). The MRS broth inoculated with *L. plantarum* (MRSp) sample had the highest TTA (0.64%) over a period of 24 h and the uninoculated MRS broth sample (MRS (C)) had the slightest increase in TTA (0.1%) within the 24 h period. After 48 h, the synthetic media 5 (M5p) sample recorded a high TTA (0.60%). The WT inoculated with *L. plantarum* (WTp) sample had a drastic decrease in TTA over time.

**Figure 5:**
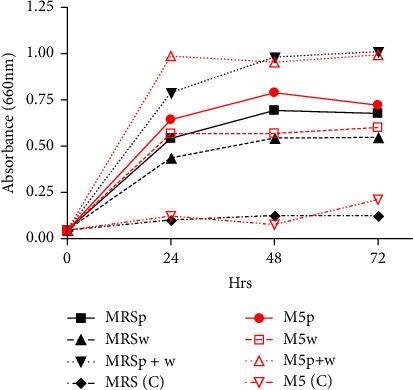
Effect of different media formulations on the growth rate of starter cultures.

As illustrated in Figure 5, there was a notable difference in absorbance between MRS broth inoculated with *L. plantarum* (MRSp) and MRS media inoculated with *W. confusa* (MRSw). The absorbance increased with time, with a peak of 0.987 recorded after 24 h for M5p + w. MRS (C) sample had the lowest absorbance as well as the minimal change in absorbance since it remained close to constant over the 72 h period.

**Figure 6:**
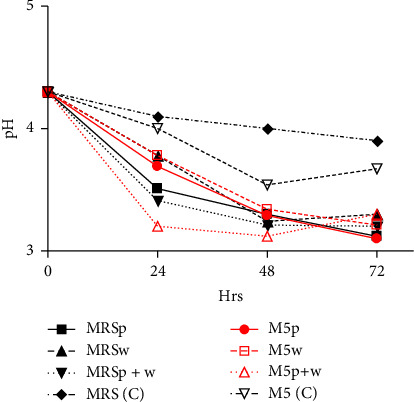
Effect of inoculating media with different starter cultures on pH.

From Figure 6, it is evident that pH drastically dropped within the first 24 h of inoculation. Thereafter, the decline in pH was gradual for all samples. The cocultured (M5p + w) media and cocultured MRS (MRSp + w) exhibited change in pH from 4.3 to 3.2 and 4.3 to 3.3, respectively, after 72 h. The uninoculated MRS (C) sample had a slight change in pH from 4.3 to 4.1. However, synthetic medium inoculated with *L. plantarum* and *W. confusa* (coculture) showed fluctuations in pH over the incubation period.

**Figure 7:**
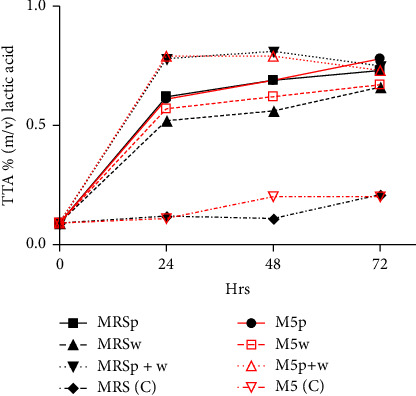
Effect of inoculating media with different starter cultures on TTA.

Titratable acidity increased over time after inoculation, as shown in Figure 7. The synthetic and culture medium (M5p + w) exhibited an increase in TTA (0.79%) within the first 24 h. As time progressed during the incubation period, MRS broth inoculated with *L. plantarum* and *W. confusa* (MRSp + w) increased gradually to a peak of 0.81% (m/v) LA.

As illustrated by Table 3, there was a significant difference (*p* > 0.05) between the survival rate of *L. plantarum* and *W. confusa* upon exposure to heat shock. *W. confusa* in synthetic media recorded a higher survival rate (27 ± 0.03%) upon exposure to heat shock than *L. plantarum* (7 ± 0.01%). In contrast, *L. plantarum* in MRS recorded a higher survival rate (67 ± 0.02%) upon exposure to cold shock and oxidative stress (34 ± 0.01%). Upon exposure to osmotic pressure, *W. confusa* recorded lower survival rates than *L. plantarum* in synthetic media.

## 5. Discussion

### 5.1. Effect of the Type of Media on the Growth of *L. plantarum*

From Figure 1, it is noted that the absorbance increased after *L. plantarum* inoculation overtime. Studies conducted by Stevenson et al. [35] also support this assertion since they found that increase in LAB concentration during the growth phase increases the absorbance. LAB are heterotrophic microorganisms and have high nutritional requirements for amino acids and vitamins [8, 36]. LAB are microorganisms fastidious from the nutritional point of view as they require some vitamins, amino acids and bases to grow [13, 37]. The nutritional requirements of LAB are complex and vary, even between strains within the same genera. The fermentation wort used in this study can support the growth of LAB given that spontaneous LA fermentation occurs naturally. The wort had sufficient carbohydrates, proteins, amino acids, fatty acids, vitamins and minerals for proper growth. The metabolic activity and fermentation efficiency of LAB depend on the availability of fructose, some amino acids (e.g., lysine, arginine and glutamic acid) and microelements (e.g., manganese, magnesium and potassium) [11, 38]. Quinoa used in medium development contains these components (i.e., sugars, vitamins, amino acids and trace elements), which might enhance the yield of the biomass. From Figure 1, it is evident that as the concentration of quinoa hydrolysate increases, so does the biomass. The use of gluten-free quinoa in medium development may be another advantage since its main proteins (globulins and albumins) are more hydrophilic than gluten and, therefore, more accessible for protease enzymes. The hydrolysis of quinoa proteins by LAB could enhance their growth and metabolic activity towards the release of organic acids, small peptides and amino acids [13]. The growth of *L. plantarum* is due to the breakdown capacity of the protein chain by LAB into amino acids and peptides which are more easily assimilated [39]. *Weissella* spp. are frequently associated with *Lactobacillus*, *Leuconotocs*, *Lactococcus* and *Pediococcus* in different habitats and in food fermentation [7].

### 5.2. Effect of Starter Cultures on pH

Measuring the pH value is one of the simplest ways to determine fermentation adequacy and is thus also a simple way to compare the performance of different strains [40]. The initial pH of the growth media was 4.5. After inoculation, as illustrated in Figure 2, there was a decrease in pH with time in all the media samples as fermentation progressed. pH decreases during fermentation due to organic acid production [41, 42]. Decrease in pH was a result of acidity, possibly due to the microbial activity on the carbohydrates and other food nutrients to produce organic acids, mainly LA. Furthermore, LAB are able to metabolize starch, leading to the drop of the pH and enhancing the production of organic acids such as lactic [14, 38]. According to literature, sour beers should contain LA in the range of 3–6 g/L and have a pH of 3.3–3.9 [38]. Peyer et al. [43] revealed that if LAB metabolism was reduced below pH 4.9 and the growth of LAB growth was completely inhibited at pH below 3.4.

However, the use of cocultures resulted in drastic decrease in pH as evidenced in Figure 6. This can be attributed to the synergistic effect of the two isolates. In addition to mainly producing LA, LAB also produce acetate, propionate, 3-hydroxypropionate, formate and succinate [22, 37, 40]. These short chain fatty acids are the result of LAB metabolism. In a related study, LA and acetic acid were the main organic acids after LA fermentation and the highest in quinoa based product was LA: 7.58 mg/mL and acetic acid: 2.23 mg/mL [44].

### 5.3. Effect of Starter Cultures on the Brix Value

The initial Brix value of the growth media was 8.5%. After a period of 24 h, the Brix values decreased. This change suggests that the fermentation process was taking place. *L. plantarum* is known to be homofermentative for hexoses, producing 2 mol LA per mol of hexoses [15, 45]. Given the lack of a functional respiratory system, LAB obtain energy through substrate-level phosphorylation following two metabolic pathways for hexose fermentation, that is, homofermentative and heterofermentative [46]. Quinoa-based media had the potential to produce fermentable sugars from hydrolysis step with LAB, converting hexose sugars almost exclusively to LA, and heterofermentative species fermenting hexose sugars to LA, CO_2_ and ethanol or acetic acid [21]. However, the efficiency of acid hydrolysis in the release of fermentable sugars used in this study is low compared with enzymatic hydrolysis. The drop in the Brix value in the synthetic growth media was due to the utilization of sugars present by LAB starter cultures. As wort ferments, the simple sugars are converted to LA. *L. plantarum* is the most common amylolytic LAB species in fermented foods, displaying high extracellular or cell-bound amylase activity [8]. The rapid utilization of sugars in the synthetic media inoculated with LAB enables them to acidify the media to a pH below 4.2; hence, decrease in the Brix value is directly proportional to the decrease in pH. The ability of starter cultures to metabolize sugars available in a substrate is an important aspect for starter culture selection [47].

### 5.4. Effect of Starter Cultures on TTA

The TTA increased with starter cultures inoculation over time (Figures 3 and 7). The titratable acidity of samples increases during the fermentation process [44]. The increase in acidity as fermentation progressed in all inoculated growth media is explained by the accelerated growth rate of LAB [37]. As shown in Figure 4, there was a sharp increase in titratable acidity during fermentation with a fairly constant TTA for the control. This observation could be explained by LA fermentation, there is conversion of glucose to LA under anaerobic conditions. In the metabolism of LAB, succinic, acetic and malic acids are also produced in the course of fermentation [22, 38]. The carbon dioxide produced by fermentation might also lead to decrease in pH [44] due to increased acidity. These acids are also responsible for the sour taste in opaque beer commonly referred to as the “bite.” The sour taste is an important sensory attribute in opaque beer production. In this study, values of 0.79% were recorded in synthetic cocultured wort at 72 h. High TTA values may indicate potential of early souring and consequently short shelf-life.

## 6. Effect of Heat Shock on the Survival of LAB

From the results (Table 3) obtained in this study, it was noted that *L. plantarum* and *W. confusa* all survived under exposure to heat (52°C) but survival rates differed since *L. plantarum* showed a lower survival rate (12%) than *W. confusa* (30%). Therefore, *W. confusa* had better tolerance to heat than *L. plantarum*. The effect of heat shock and the induction of a stress response in *Lactobacillus* spp. have been studied [18]. Overall, heat shock resistance of bacteria is affected by genetic differences among species, the physiological status of the cells and environmental factors, such as pH, water activity, salt content and preservatives [48]. Bacterial responses to high temperatures is caused by a set of heat-inducible proteins known as heat shock proteins (HSPs), which are employed to counteract the pleiotropic effects of heat stress. Heat resistance in *L. plantarum* induces the synthesis of proteins which have various roles in cell physiology, including chaperone activity, ribosome stability, stringent response mediation, temperature sensing and control of ribosomal function [48]. Major HSPs partake in the repair and turnover of damaged proteins. At an increased environmental temperature, not only the expression of stress proteins is increased in LAB cells but also the capacity of chaperones binding to unfolded polypeptides demonstrates substantial improvement cells [49]. Heat stress is commonly encountered by many LAB during food fermentation, especially high temperatures (> 60°C) used for pasteurization. The *Lactobacillus* has multiple defence mechanisms to cope with various stress conditions, including preserving cell energy and changing cell membrane compositions [50, 51].

## 7. Effect of Cold Shock on the Survival of LAB

Results in Table 3 showed that low temperatures have significant (*p* < 0.05) effect on survival rate of these bacteria. Studies conducted by Fusco et al. [52] also stipulate to this assertion since it was concluded that *Lactobacillus* spp and *Weisella* spp can tolerate temperatures of 0°C. The survival of LAB upon low temperatures is mainly due to the induction of a set of proteins called cold-shock proteins (CSPs) due to cold stress [53]. Small acidic CSPs are known to be highly induced in response to cold shock [24]. Three *csp* genes (*cspL*, *cspC* and *cspP*) were identified in *L. plantarum* [24, 54]. Cold-shock response is classically exhibited when an exponentially growing culture is shifted from its optimum growth temperature to a lower temperature [55]. Cold temperatures above freezing may lead to growth arrest of LAB, but such conditions do not abruptly provoke cell death. It is well established that after a quick downshift in temperature (cold shock), a set of proteins are preferentially expressed, among which a set of small-barrel proteins referred to as the major CSPs show the highest induction level [23].

In fact, many LAB can be stored at low temperatures (> 0°C) for several days. In contrast, freezing of LAB cultures influences survival in a strain-dependent manner [52]. Lactobacilli are naturally equipped with a plethora of defence mechanisms, such as chaperone proteins (Gro ES/GroEL and DnaK/DnaJ/Grp E), proteases, transport systems and proton pumps to enhance survival in stressful environments [55]. However, cold stress would cause changes in membrane fluidity (influencing nutrient transport), lowering of enzyme activity (influencing cellular metabolism) and RNA structure stability (influencing translation process), all of which further influence the bacteria viability. The results of a study by [56] revealed that *L. plantarum* K25 displayed a complex biological network to tackle cold stress mainly by adjusting carbohydrate, amino acid and fatty acid metabolism and biosynthesis.

### 7.1. Effect of Osmotic Pressure on the Survival of LAB

Table 3 shows the results in terms of growth rate of LAB after determining the optical density (absorbance) of cells with 25% (v/v) NaCl concentration as osmotic pressure components. Increase on the osmotic pressure due to increase in NaCl concentration causes a significant (*p* < 0.05) decrease in the growth rate of LAB. Decrease in LAB concentration due to increase in sodium chloride is mainly due to osmotic stress which decreases the positive turgor of bacterial cells as a result of dehydration.

### 7.2. Effect of Oxidative Stress on the Survival of LAB

The survival rate of LAB after treatment with L-cysteine and L-ascorbic acid as oxygen-scavenging components was also assessed (Table 3). The bacterial isolates showed a significant difference in survival rates in both MRS and synthetic media. This could be attributed to the phenolic compounds in the quinoa-based medium. Rodríguez et al. [57] reported that *L*. *plantarum* was able to increase antioxidant activity and improve food aroma compounds by the degradation of some food phenolic compounds through the metabolic activity of LAB [39]. During fermentation of quinoa flour*, L. plantarum* T6B10 allowed the increase of the antioxidant and phytase activities and the degradation of condensed tannins [58]. *W. confusa* has been used as a starter for different kind of fermented foods for its ability to produce high amounts of dextran and modify the texture in cereals [58]. In synthetic media, *L. plantarum* had relatively higher survival rates (32 ± 0.01%) than *W. confusa* (15.05 + 0.07%). The ability of *L. plantarum* to modulate antioxidant activity and phenolic compound composition appears strain dependent but also dependent on the matrix investigate [59]. The availability of probiotic microorganisms as natural antioxidants has also been investigated [60]. However, in a related study, *Weissella* spp. strains had consistently higher antioxidant activity through changing their amino acid profiles [61]. LAB such as *W. confusa*, *L. plantarum*, *L. rhamnosus* and *L. mesenteroides* have been proved to be capable of reducing free radicals. Katina et al. [62] showed that the levels of folates, free phenolic acids, total phenolic compounds, lignans and alkylresorcinols were increased through fermentation. In this study, the survival rates after oxidative stress were relatively low. Since endogenous antioxidant capacity is limited, exogenous supplementation is required to regulate the oxidative stress [63, 64].

## 8. Conclusion

*L. plantarum* and *W. confusa* can be used as LAB starter cultures to produce LA in the quinoa-based medium. The quinoa-based synthetic media achieved higher concentration of bacterial cells than the commercial wort. The WS achieved higher LAB growth than the WT. *L. plantarum* and *W. confusa* showed stability under different stress conditions and concentration. The use of cocultures in this study resulted in favourable biochemical characteristics than monocultures. This synergistic effect may improve the keeping qualities of opaque beer and, consequently, improve the sensory attributes. *L. plantarum* and *W. confusa* are good candidates for industrial production of opaque beer with tolerances to heat, shock and osmotic pressure. Further studies can elucidate the effect of inoculum dose and other fermentation variables using predictive mathematical modelling.

## Figures and Tables

**Table 1:** Quantities of the components used to prepare the synthetic media.

Media	Quinoa hydrolysate % (v/v)	Calcium carbonate (g)	Magnesium chloride (g)	Sucrose (g)	Water % (v/v)	Whey milk % (v/v)	Vegetable oil % (v/v)
Medium 1	15	3	3	4	85	—	—
Medium 2	39	3	3	4	50	20	1
Medium 3	44	3	3	4	35	20	1
Medium 4	59	3	3	4	20	20	1
Medium 5	74	3	3	4	5	20	1

**Table 2:** Inoculation protocol for lactic acid fermentation using *L. plantarum (p)* and *W. confusa (w)*.

Sample ID	Inoculation protocol
MRSp	MRS broth inoculated with *L. plantarum*
MRSw	MRS broth inoculated with *W. confusa*
MRSp + w	MRS broth inoculated with *L. plantarum* and *W. confusa* (coculture)
MRS (C)	MRS broth uninoculated (control)
WTp	Tyndallized opaque beer wort inoculated with *L. plantarum*
WSp	Sterilize opaque beer wort inoculated with *L. plantarum*
M1p	Synthetic medium 1 inoculated with *L. plantarum*
M2p	Synthetic medium 2 inoculated with *L. plantarum*
M3p	Synthetic medium 3 inoculated with *L. plantarum*
M4p	Synthetic medium 4 inoculated with *L. plantarum*
M5p	Synthetic medium 5 inoculated with *L. plantarum*
M5p + w	Synthetic medium 5 inoculated with *L. plantarum* and *W. confusa* (coculture)
M5c	Synthetic medium 5 inoculated with *W. confusa*

**Table 3:** Effect of various stress conditions on the survival rate of starter cultures.

Strain	Inoculums preparation conditions			Survival rate after heat shock (%)	Survival rate after cold shock (%)	Survival rate after osmotic pressure (%)	Survival rate after oxidative stress (%)
	Temp °C	Media	pH				
*L. plantarum*	37	MRS	3.84	12.03 ± 0.014^a^	67.02 ± 0.014^a^	23.04 ± 0.057^a^	34.15 ± 0.212^a^
*W. confusa*	37	MRS	4.05	30.04 ± 0.028^b^	51.01 ± 0.014^b^	19.10 ± 0.141^b^	22.02 ± 0.028^b^
*L. plantarum*	37	Synthetic	4.00	7.01 ± 0.014^c^	53.03 ± 0.014^c^	25.03 ± 0.042^c^	32.01 ± 0.014^c^
*W. confusa*	37	Synthetic	4.10	27.03 ± 0.042^d^	47.01 ± 0.014^d^	11.01 ± 0.014^d^	15.05 ± 0.071^d^

*Note:* Values are the mean ± standard deviation of triplicate determinants. Mean values (*n* = 3) within the same column bearing different superscripts differ significantly (*p* < 0.05).

## Data Availability

The dataset used to support the findings of this study is available on request from the corresponding author.
